# Cohort analysis of novel *SPAST* variants in SPG4 patients and implementation of *in vitro* and *in vivo* studies to identify the pathogenic mechanism caused by splicing mutations

**DOI:** 10.3389/fneur.2023.1296924

**Published:** 2023-12-07

**Authors:** Rosangela Ferese, Simona Scala, Antonio Suppa, Rosa Campopiano, Francesco Asci, Alessandro Zampogna, Maria Antonietta Chiaravalloti, Annamaria Griguoli, Marianna Storto, Alba Di Pardo, Emiliano Giardina, Stefania Zampatti, Francesco Fornai, Giuseppe Novelli, Mirco Fanelli, Chiara Zecca, Giancarlo Logroscino, Diego Centonze, Stefano Gambardella

**Affiliations:** ^1^IRCCS Neuromed, Pozzilli, Italy; ^2^Department of Human Neurosciences, Sapienza University of Rome, Rome, Italy; ^3^Genomic Medicine Laboratory, IRCCS Fondazione Santa Lucia, Rome, Italy; ^4^Department of Translational Research and New Technologies in Medicine and Surgery, University of Pisa, Pisa, Italy; ^5^Department of Biomedicine and Prevention, University of Rome “Tor Vergata”, Rome, Italy; ^6^Department of Biomolecular Sciences, University of Urbino “Carlo Bo”, Urbino, Italy; ^7^Center for Neurodegenerative Diseases and the Aging Brain, Department of Clinical Research in Neurology of the University of Bari “Aldo Moro” at “Pia Fondazione Card G. Panico” Hospital Tricase, Lecce, Italy; ^8^Department of Systems Medicine, Tor Vergata University, Rome, Italy

**Keywords:** *SPAST*, minigene assay, diagnosis, neurogenetics, rare disease

## Abstract

**Introduction:**

Pure hereditary spastic paraplegia (SPG) type 4 (SPG4) is caused by mutations of *SPAST* gene. This study aimed to analyze *SPAST* variants in SPG4 patients to highlight the occurrence of splicing mutations and combine functional studies to assess the relevance of these variants in the molecular mechanisms of the disease.

**Methods:**

We performed an NGS panel in 105 patients, *in silico* analysis for splicing mutations, and *in vitro* minigene assay.

**Results and discussion:**

The NGS panel was applied to screen 105 patients carrying a clinical phenotype corresponding to upper motor neuron syndrome (UMNS), selectively affecting motor control of lower limbs. Pathogenic mutations in *SPAST* were identified in 12 patients (11.42%), 5 missense, 3 frameshift, and 4 splicing variants. Then, we focused on the patients carrying splicing variants using a combined approach of *in silico* and *in vitro* analysis through minigene assay and RNA, if available. For two splicing variants (i.e., c.1245+1G>A and c.1414-2A>T), functional assays confirm the types of molecular alterations suggested by the *in silico* analysis (loss of exon 9 and exon 12). In contrast, the splicing variant c.1005-1delG differed from what was predicted (skipping exon 7), and the functional study indicates the loss of frame and formation of a premature stop codon. The present study evidenced the high splice variants in SPG4 patients and indicated the relevance of functional assays added to *in silico* analysis to decipher the pathogenic mechanism.

## 1 Introduction

Hereditary spastic paraplegias (HSPs) are inherited motor neuron disorders caused by mutations that may occur in more than 70 distinct loci (SPG1–72). These mutations vary concerning inheritance patterns, including pure and complicated autosomal dominant, autosomal recessive, and X-linked chromosomes (1, 2).

The most frequent HSPs are autosomal dominant hereditary spastic paraplegia (ADHSPs), with a prevalence ranging from 1.8 to 5.5/100,000 in most populations (3, 4). Pure hereditary spastic paraplegia (SPG) type 4 (SPG4) is the most common form, accounting for 15–40% of all HSP cases (3, 5–10). Patients affected by SPG4 manifest an upper motor neuron syndrome, which is characterized by weakness mostly involving the lower limb and increased muscle tone (i.e., spasticity) involving both proximal and distal muscles. Moreover, neurogenic urinary disturbances, including urgency, hesitancy, and incontinence, are usually present. According to this clinical syndrome, SPG4 is defined as a non-complicated or pure HSP. Such a definition rules out those syndromes, including ataxia, dementia, developmental delay, optic neuropathy, retinopathy, peripheral neuropathy, amyotrophy, extrapyramidal dysfunction, deafness, or ichthyosis (11).

SPG4 is caused by mutations in *SPAST* (located on 2p22.3), which encodes the microtubule-severing protein spastin, a member of the AAA (ATPase associated with various cellular activities) protein family. Hundreds of variants without mutational hotspots have been reported, including missense mutations clustered mainly in the AAA domain, along with nonsense, splice-site point mutations, insertions, and deletions found in all regions (6, 8, 12–16).

Next-generation sequencing (NGS) represents the best approach for the genetic study of HSP since it allows a massive concomitant analysis of more than 80 causal SPG genes (1, 17), which allow a detection rate between 20 and 70% considering sporadic vs. familial patients in diagnostic procedures (18).

Variants in the *SPAST* gene are the most common cause of HSP and, depending upon the ethnic background of patients, account for 15–40% of all HSP cases (7–9).

Among *SPAST* mutations, splicing events occur roughly in 10% of patients with a pathogenic variant (12) although it is likely that such a prevalence is underestimated due to the sequencing pipeline used in routine NGS experiments for current molecular diagnosis. This approach allows routine detection of canonical splicing variants only. These correspond to mutations falling within essential dinucleotide sites, while other splicing variants (i.e., deep intronic, near splice-site, synonymous, or missense) are either missed out or cannot be detected unless additional studies are carried out, and their molecular relevance is established (19).

The present study aimed to detect *SPAST* variants in SPG4 patients, deciphering the aberrant molecular mechanisms that lead to the disease state. This is carried out by implementing a panel including 80 genes involved with HSP. The study was conducted on 105 patients with a clinical phenotype compatible with an upper motor neuron syndrome (UMNS) selectively affecting lower limbs. The NGS was implemented by molecular approaches to detect pathogenicity and molecular mechanisms of novel splicing variants.

## 2 Materials and methods

### 2.1 Patients

All patients were recruited by IRCCS Neuromed Institute, Pozzilli (IS), Department of Human Neurosciences, Sapienza University of Rome and Center for Neurodegenerative Diseases at Fondazione Panico, Tricase (LE), according to the following inclusion criteria: (1) clinical phenotype compatible with upper motor neuron syndrome (UMNS) and selectively affecting lower limbs; (2) exclusion of sporadic, non-genetic causes of paraplegia (e.g., brain and spinal cord lesions) as detected from 1.5T MRI scanning. Once recruited, all patients were scored by applying the spastic paraparesis rating scale (SPSR), the modified Ashworth scale (MAS), and Barthel Index (BI). The instrumental evaluation of patients included measurement of nerve conduction, somatosensory-evoked potentials (SSEP), and motor-evoked potentials (MEP) both to the upper and lower limbs. All patients gave written informed consent in agreement with the Helsinki Declaration. The local ethical committee approved the study.

The study cohort included 105 patients, already considered in Ferese et al. (20) focusing on non-canonical splice variants. In the present study, the same cohort of patients has been well described and characterized, and the focus was shifted to canonical splice site mutations.

### 2.2 DNA extraction

Genomic DNA was isolated from peripheral blood leukocytes according to standard procedures (QIAamp DNA Blood Mini Kit–QIAGEN).

### 2.3 Next-generation sequencing panel

The NGS analysis was performed using the SeqCap EZ Choice Enrichment Kits (Hoffmann-La Roche, Basel) on an Illumina MiSeq (San Diego, CA). A full list of genes sequenced is provided in Supplementary Table 1. All coding exons of the RefSeq transcripts of the genes and 15 base pairs of the flanking introns were targeted. In total, 99% of the coding exons were sequenced with a minimal read depth of 30X.

GenomeUp software (https://lab.juliaomix.com/) was used for data analysis. It provides automated annotation (Best Practices workflows of GATK v4.1 for germline variant calling), alignment of sequence reads to the reference genome GRCh37/hg19, and selection of potentially pathogenic variants. Direct evaluation of data sequence was performed by the Integrative Genomics Viewer v.2.3. Mutation re-sequencing and segregation analysis were performed by the Sanger sequencing ABI 3130xl Genetic Analyzer (Applied Biosystems).

### 2.4 Data analysis and variants interpretation

Variants were classified with the help of public databases (VarSome https://varsome.com; GnomAD https://gnomad.broadinstitute.org) and according to the American College of Medical Genetics Guideline for germline variant classification (pathogenic (class 5), likely pathogenic (class 4), and variants of uncertain significance (VoUS; class 3) (21). *In silico* analyses were performed using SIFT, PolyPhen, PROVEAN, and Mutation Assessor. The novel variants identified have been submitted to the ClinVar database.

### 2.5 *In silico* analysis for splicing mutations

The identified DNA variation was tested for potential splicing effects using the following online software products: varSEAK SSP (https://varseak.bio) is a website that provides information about genetic variants from public databases. The prediction of how a splicing site may lead to functional (positive values) or non-functional (negative values) effects was expressed by a probability score ranging from −100% to +100%. This is expressed by ΔScore (DeltaScore): the difference between the score of the splice site on the reference sequence and the score of the splice site on the variant sequence. NNSPLICE (http://www.fruitfly.org/seq_tools/splice.html) employs two “neural networks” that were trained on consensus splice sites while also considering dinucleotide frequencies due to the strong correlation between neighboring nucleotides in splice site consensus sequences (22), NNsplice assigns a score of 0 to 1 for native splice sites EX SKIP (https://ex-skip.img.cas.cz). This software compares the exonic splicing enhancers (ESEs) vs. exonic splicing silencers (ESSs) profile of a wild-type and a mutated allele to quickly determine which exonic variant has the highest chance to skip this exon. The software calculates the total number of ESSs, ESEs, and their ratio. Specifically, it computes the number of RESCUE-ESEs (23), fluorescence-activated screen for exonic splicing silencers (FAS-ESSs) (24), putative ESEs (PESEs) on putative ESSs (PESSs) (25), neighborhood inference (26), and exon-identity elements (EIE) on intron-identity elements (IIEs) (27) for each segment. CRYP-SKIP (https://cryp-skip.img.cas.cz) compares the ESE/ESS profile of a wild-type and a mutated allele to quickly determine which exonic variant has the highest chance to skip this exon. SpliceRover (http://bioit2.irc.ugent.be/rover/splicerover) is a prediction tool that can be used for donor and acceptor splice site prediction to gives us a score, the median for donor splice sites increases from 0.816 to 0.907 score (28).

### 2.6 *In vitro* splicing analysis (minigene assay)

The potential splicing effect of the *SPAST* variants was investigated by using the pSPL3 minigene vector (exon trapping system, Gibco, BRL, Carlsbad, CA). The *SPAST* exon and flanking intronic sequences were amplified using the DNA from heterozygous patients as a template.

In case of an intron is too small, the assay requires the insertion of two exons. Instead, it is not possible to perform this assay when the variant is localized in the last exon since the splicing junction is not present.

The minigene constructs containing either the wild-type or variant sequence were transfected into HEK 293 cells by Lipofectamine 2000 (Invitrogen Corporation, Carlsbad, CA). After 48 h, total cellular RNA was isolated with the acidic guanidine phenol-chloroform method. First-strand cDNA was synthesized by SuperScript^®^ VILO^TM^ (Thermo Fisher Scientific). RT-PCR was performed using vector exonic primers SD6 (forward) and SA2 (reverse) according to the manufacturer's instructions. The final PCR products obtained from transfection with wild-type and variant plasmids were analyzed by DNA sequencing.

### 2.7 Literature review

**Figure 1:**
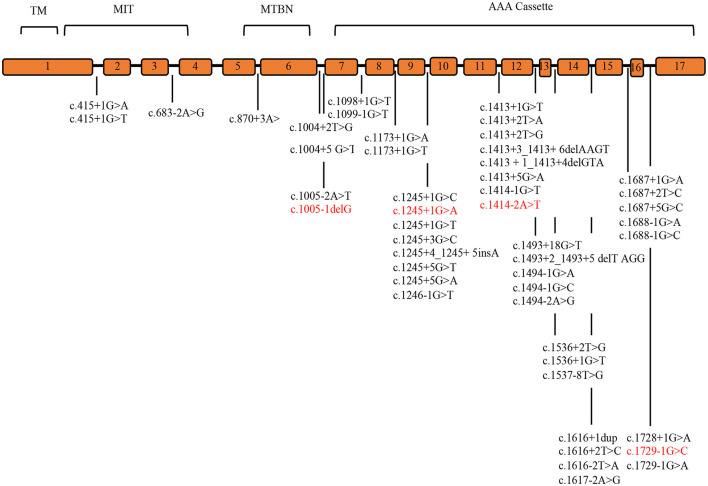
*SPAST* splicing variants reported in the literature. The *SPAST* gene spans the region of ~90 kb of genomic DNA and contains 17 exons. Mutations detected in this study are shown in red. TM (57–79 amino acids), MIT (116–197 amino acids), MTBD (270–328 amino acids), and AAA cassette (342–599 amino acids) are highlighted.

Systematic literature review was conducted to identify the detection rate of genetic variants and the clinical phenotype of SPAST patients. Pubmed, Medline, and Embase databases identified 20 cohort analysis studies consisting of world SPG patients between 2000 and 2022. The literature studies identified 48 splicing mutations, 83.3% of which are placed in the AAA Cassette (40/48), while 6.2% (3/48) are placed in the MIT motive, and 10.4 % (5/48) in the MTBN motive. These data are summarized in Supplementary Table 2 and Figure 1 (8, 11, 15, 17, 20, 24, 29–43).

## 3 Results

### 3.1 Cohort and genetic analysis

The study cohort included 105 patients, all suffering from upper motor neuron syndrome affecting lower limbs. In detail, patients manifested a variable severity of lower limb weakness, predominant in the distal muscles, associated with hyperreflexia, sustained ankle clonus, and finally, Babinski's sign (i.e., spasticity). None of these patients suffered from sensory loss, cerebellar dysfunction, or additional neurological signs and symptoms, which are commonly observed in HSP-plus conditions, as reflected by the spastic paraparesis rating scale (SPRS) scores. None of these patients had a concomitant involvement of the peripheral nervous system, as shown by normal nerve conduction studies. All patients had normal SEP from both upper and lower limb stimulation. In this cohort of SPG4 patients, MEP was typically normal when measured at the level of the upper limbs, whereas a variable degree of impairment was recorded from lower limb muscles.

A total of 12 patients have pathogenic variants in SPG4, 2 in SPG7, 1 KIF5a, 1 in SPG11.

Among patients with *SPAST* variants, 5 carry a missense, 3 a frameshift, and 4 carry splicing variant (Table 1) (20). These variants were identified in three familial and eleven sporadic patients (four have been previously reported as causative of HSPs, and nine are novel variants). All variants, except p.Lys90Ter (TM domain) and p.Lys236ProfsTer6 (MTBN domain), are placed in the AAA Cassette. Nine of these variants are novel, while four have been previously reported p.Arg460Cys (44), c.1245+1G>A (11), and p.Arg431Ter (12). These variants are classified as class 4 or 5, according to ACMG guidelines.

**Table 1:** Genetic and clinical data in the cohort of patients analyzed.

**Family ID**	**HGVSc**	**HGVSp**	**dbSNP ID**	**Mutation**	**ACMG**	**Criteria**	**Clinvar**	**Genotype**	**Age**	**Onset**	**Sex**	**Family history**	**Score**	**Ref**
25	c.1378C>T	p.Arg460Cys	rs878854990	Missense	5	PM1-PM2-PM5-PP2-PP3-PP5	SCV000290032	Hz	45	33	F	YES	151.38.00	(44)
71	c.1679C>T	p.Pro560Leu		Missense	4	PM1-PM2-PP2-PP3	SCV000492797	Hz	65	44	M	YES	161.26.00	This study
159	c.1245 + 1G>A			Splicing	5	PVS1-PP5-PM2-PP3	SCV001745884	Hz	55	35	F	YES	165.41.00	(32)
497	c.1414-2A>T			Splicing	5	PVS1-PM2-PP3	SCV001745882	Hz	57	47	F	NO	165.41.00	This study
246	c.268A>T	p.Lys90Ter		Missense	5	PVS1-PM2	Submitted	Hz	51	35	M	NO	150.57.00	This study
565	c.1215_1219del	p.Asn405LysfsTer36	rs1553317032	Frameshift, Ter	5	PVS1-PM2-PP3-PP5	SCV000645342	Hz	51	40	M	NO	134.53.00	(17)
861	c.1729-1G>C			Splicing	5	PVS1-PM2-PP3	SCV001745878	Hz	68	NA	F	NO	165.41.00	This study
879	c.1774_1775insG	p.Ile592SerfsTer39		Frameshift, Ter	5	PVS1-PM2-PP3	SCV001451018	Hz	36	30	F	YES	122.15.00	This study
992	c.1349G>A	p.Arg450Lys		Missense	4	PM5-PM2-PM1-PP3	SCV002061744	Hz	59	6	M	YES	160.19.00	This study
1281	c.1291C>T	p.Arg431Ter	rs786204126	Missense	5	PVS1-PM2-PP3-PP5	SCV000218741	Hz	79	NA	F	NO	143.19.00	(12)
1323	c.1005-1delG			Splicing	5	PVS1-PM2-PP	SCV001745879	Hz	73	53	M	YES	128.43.00	This study
1823	c.706_710del	p.Lys236ProfsTer6		Frameshift, Ter	5	PVS1-PM2	Submitted	Hz	67	NA	M	NO	121.35.00	This study

NA, not applied; Hz, heterozygous; F, female; M, male.

### 3.2 Splicing mutations

Four splicing variants have been identified in four families (Table 2).

**Table 2:** *In silico* prediction analyzes.

**Family ID**	**HGVSc**	**ACMG 1**	**Clin Var**	**Splicing class**	**varSEAK SSP**	**NNSPLICE**	**EX SKIP**	**CRYP-SKIP**	**SPLICE ROVER Score**	***In vitro* assay**	***In vivo* assay**	**ACMG 2**
159	c.1245 + 1G>A	Class 5 (PVS1-PP5-PM2-PP3)	SCV001745884	5	ES	LD	ES	NE	0.963	Loss exon 9	NA	Class 5 (PVS1-PP5-PM2-PP3-PS3)
497	c.1414-2A>T	Class 5 (PVS1-PM2-PP3)	SCV001745882	5	LF	LD	ES	ES	0.951	Loss exon 12	NA	Class 5 (PVS1-PM2-PP3-PS3)
1323	c.1005-1delG	Class 5 (PVS1-PM2-PP3)	SCV001745879	5	LF	LD	ES	ES	0.82	Loss 8 nt exon 7	NA	Class 5 (PVS1-PM2-PP3-PS3)
861	c.1729-1G>C	Class 5 (PVS1-PM2-PP3-PP5)	SCV001745878	5	ES	NE	ES	NE	0.972	NA	NA	Class 5 (PVS1-PM2-PP3-PP5)

ES, Exon skipping; LF, Loss of function; NE, No splicing effect; LD, Loss donor site; NA, not applied; ACMG1 classification before *in vitro* and/or *in vivo* assay, ACMG2 classification after *in vitro* and/or *in vivo* assay.

#### 3.2.1 Family 497

**Figure 2:**
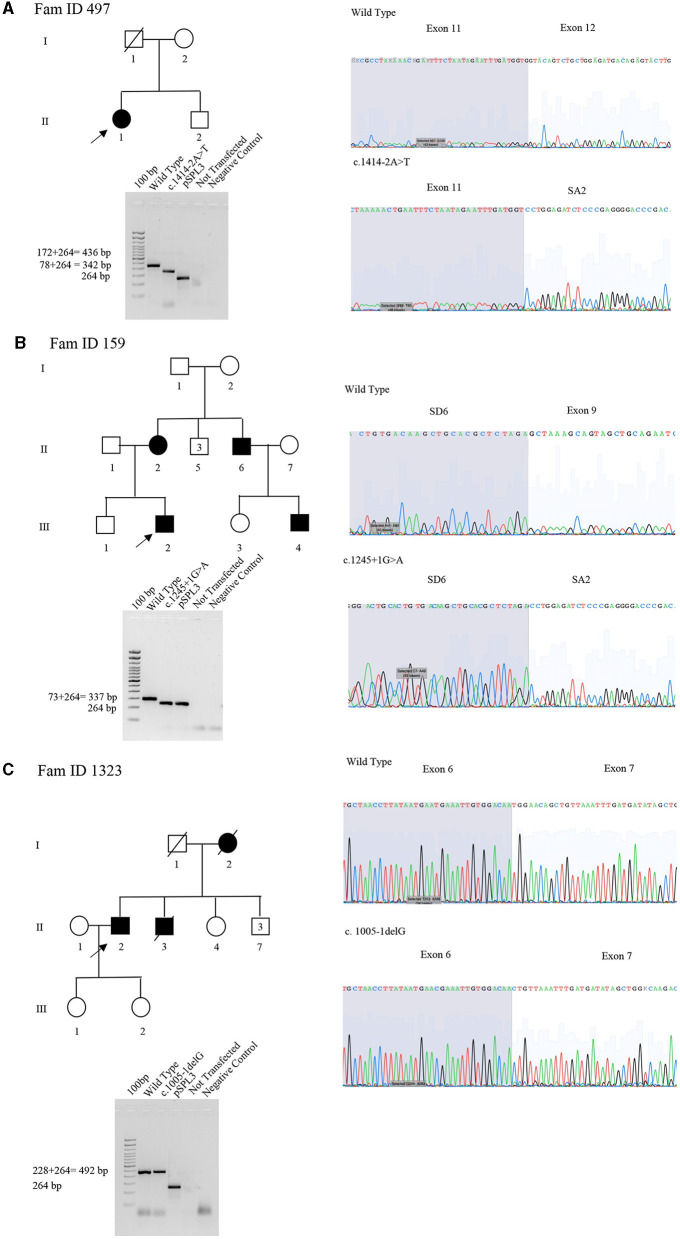
Pedigree, minigene assay, Sanger sequencing of *SPAST* families 497, 159, and 1,323. **(A)** Family ID 497. Agarose gel shows RT-PCR results of minigene assay for variant c.1414-2A>T. In lane 1 is shown an amplicon of 436 bp correspondent to a wild-type genotype (172 bp of normal splicing of exons 11 and 12 + 264 bp of pSPL3 exon); in lane 2 is shown an amplicon of 358 bp correspondent to abnormal splicing produced by variant c.1414-2A>T (358 bp [80 bp of normal splicing (exon 11)−92 bp of exon 12 loss] + 264 bp of pSPL3 exon); in lane 3 is shown the amplification of pSPL3 without *SPAST* cloning; in lane 4 is shown the amplification of HEK 293 T cDNA without transfection of pSPL3; and in lane 5 is shown negative control of PCR amplification. Sanger sequence shows the loss of exon 11 and normal sequence. **(B)** Family ID 159. Agarose gel shows RT-PCR results of minigene assay for variant c.1245+1G>A. In lane 1 is shown an amplicon of 337 bp correspondent to a wild-type genotype (74 bp of normal splicing of exon 9 + 264 bp of pSPL3 exon); in lane 2 is shown an amplicon of 264 bp correspondent to abnormal splicing produced by variant c.1245+1G>A (264 bp [74 bp of normal splicing (exon 9) – 74 bp (exon 9)] + 264 bp of pSPL3 exon); in lane 3 is shown the amplification of pSPL3 without *SPAST* cloning; in lane 4 is shown the amplification of HEK 293 T cDNA without transfection of pSPL3; and in lane 5 is shown negative control of PCR amplification. Sanger sequence shows the loss of exon 9 in *SPAST* gene and normal sequence. **(C)** Family ID 1323. Agarose gel shows RT-PCR results of minigene assay for variant c.1005-1delG. In lane 1 is shown an amplicon of 492 bp correspondent to a wild-type genotype (228 bp of normal splicing of exons 6 and 7 + 264 bp of pSPL3 exon); in lane 2 is shown an amplicon apparently of 492 bp correspondent to normal splicing produced by variant c.1005-1delG (492 bp [134 bp of normal splicing (exons 6) – 94 bp (exon 7)] + 264 bp of pSPL3 exon); in lane 3 is shown the amplification of pSPL3 without *SPAST* cloning; in lane 4 is shown the amplification of HEK 293 T cDNA without transfection of pSPL3; in lane 5 is shown negative control of PCR amplification. Sanger sequence shows the loss of eight nucleotides of exon 7 causing the loss of frame and a premature stop codon.

**Figure 3:**
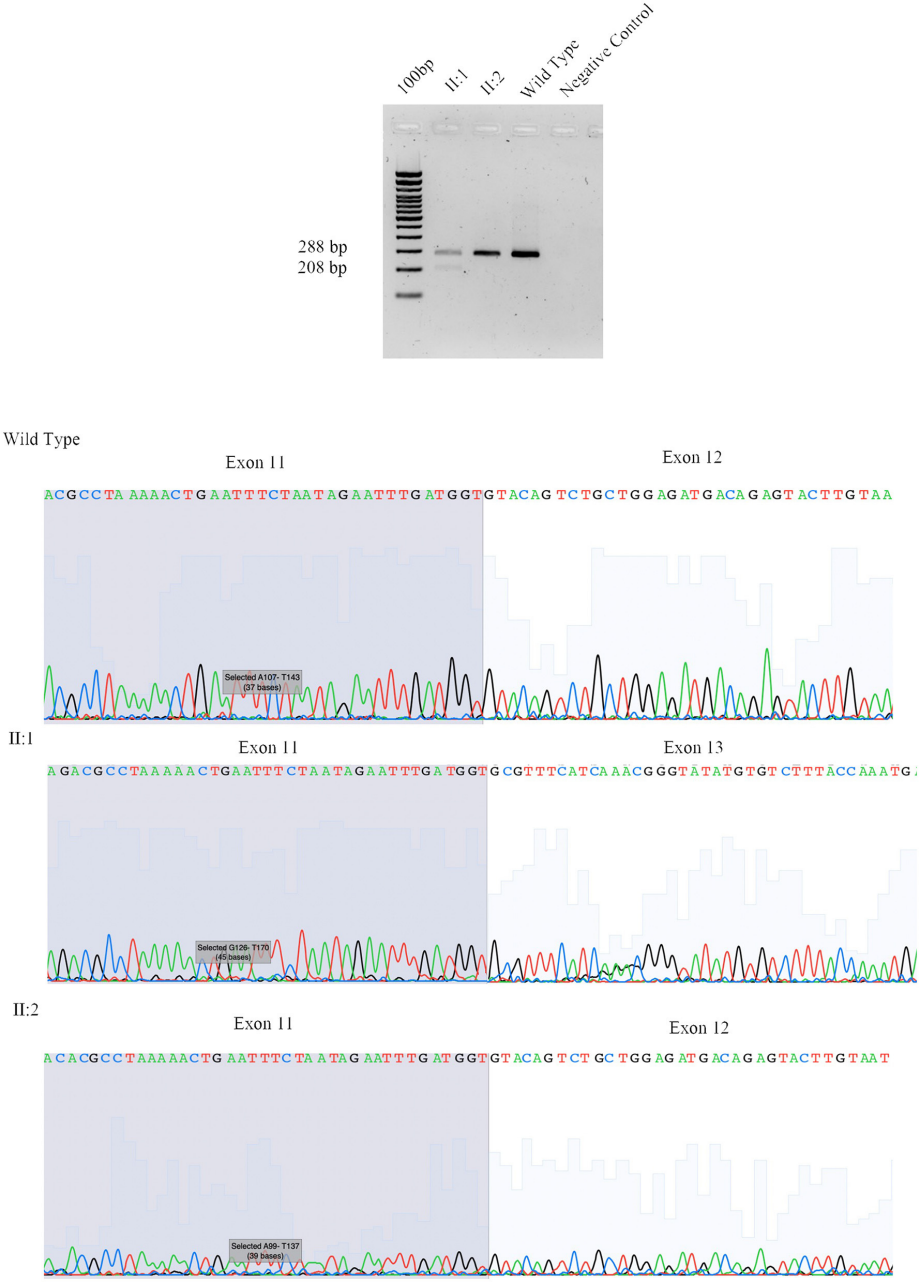
Sanger sequencing of *SPAST* family 497. We performed RNA analysis from peripheral blood of Fam ID 497: II:1 and II:2. The line 1 show the amplification from the exon 10 to exon 13 in II:1 with two amplicon of 288 bp and 208 bp corresponding at wilde-type allele and loss of exon 12, line 2 and 3 show the amplification of II:2 and wild-type sample (288 bp). The 4 line shows negative control of PCR amplification.

The proband (II:1), 57 years old female, with disease onset at 47 years, presented with progressive weakness and spasticity of the lower limbs and dysphagia. Molecular analysis identified a novel heterozygous variant c.[1414-2A>T] classified as pathogenic (PVS1-PM2-PP3). This splicing variant falls within the typical essential dinucleotides site, and it is considered a “conventional splice-site mutation.” *In silico* analysis suggests a loss of acceptor site (varSEAK SSP, NNSPLICE, EX SKIP, and CRYP-SKIP) (Table 2). This *in silico* prediction was confirmed by minigene assay, as shown in Figure 2A. PCR of cDNA without the variant produces an amplicon of 436 bp related to a wild-type genotype (172 bp of normal splicing of exons 11 and 12 + 264 bp of pSPL3 exon), while PCR of cDNA with the variant produces an amplicon of 358 bp, which indicates abnormal splicing causing the loss of exon 12 (264 + 92 bp [exon 11]). These data have been confirmed by RNA analysis *in vivo* on proband II:1 and her brother II:2 healthy (Figure 2A). The PCR analyses produce an amplicon of 288 bp (from exon 10 to exon 13) for II:2 and wild-type control, and one amplicon of 288 bp (wild-type allele) and one of 208 bp (loss of exon 12) in the proband (II:1) (Figure 3).

This was confirmed by the Sanger sequence.

#### 3.2.2 Family 159

The proband (III:2), 55-year-old female, with disease onset at 35 years, presented with progressive weakness and spasticity of the lower limbs and bladder disturbances. Molecular analysis identified a heterozygous variant c.[1245+1G>A] as previously described by McDermott et al. (32), and classified as pathogenic (PVS1-PP5-PM2-PP3). This variant falls within the essential dinucleotides site, and it is considered a “conventional splice-site mutation.” *In silico* analysis suggests a loss of donor site (varSEAK SSP, NNSPLICE, and EX SKIP) or no splicing effect (CRYP-SKIP) (Table 2). These data were confirmed by minigene assay, as shown in Figure 2B. PCR of cDNA without variant produces an amplicon of 337 bp related to a wild-type genotype (73 bp of normal splicing of exon 9 + 264 bp of pSPL3 exon), while PCR of cDNA with variant produces an amplicon of 264 bp evidencing abnormal splicing causing the loss of exon 9 (337–73 bp [exon 9]). Family members were not available for testing.

#### 3.2.3 Family 1323

The proband (II:2), 73-year-old male, with disease onset at 53 years, presented with progressive weakness and spasticity of the lower limbs, autonomic involvement with urinary and fecal incontinence, moderate hypo-pallesthesia, and severe subcortical atrophy. Molecular analysis identified a novel heterozygous variant c.[1005-1delG], classified as pathogenic (PVS1-PM2-PP3). This variant falls within the typical essential dinucleotides site, and it is considered a “conventional splice-site mutation.” *In silico* analysis suggests a loss of acceptor site (varSEAK SSP, NNSPLICE, EX SKIP, and CRYP-SKIP) (Table 2). In contrast to *in silico* predictions, the minigene assay is shown in Figure 2C. PCR of cDNA without variant produces an amplicon of 492 bp related to a wild-type genotype (228 bp of normal splicing of exons 6 and 7 + 264 bp of pSPL3 exon), while PCR of cDNA with variant producesan amplicon of 492 bp evidencing normal splicing. Instead, the sequencing analysis shows a loss of eight nucleotides of exon 7 that causes the loss of frame and a premature stop codon. Family members were not available for testing, and it was not possible to perform RNA analysis.

#### 3.2.4 Family 861

The proband, 68-year-old female, presented with pyramidal signs and progressive weakness and spasticity of the lower limbs. Molecular analysis identified a novel heterozygous variant c.[1729-1G>C] (rs1064793976) classified as pathogenic (PVS1-PM2-PP3). *In silico* analysis suggests a loss of acceptor site (varSEAK SSP, NNSPLICE, EX SKIP, and CRYP-SKIP) (Table 2). These findings could not be tested by minigene assay because the variant is present on the last exon of the gene. Moreover, we could not analyze RNA since family members were unavailable for testing.

## 4 Discussion

This study identified pathogenic *SPAST* variants in 12 out of 105 patients (11.42%, 5 missense, 3 frameshifts, and 4 splicing variants). Eight of these variants are novel, and three have been previously reported.

The rate of HSP patients with genetic mutations ranges from 50% in AD-HSP to 25% in AR-HSP, and these variations are bound to sporadic or familial patients, variability in the population, variability of clinical phenotypes, and diagnostic criteria (1). Since *SPAST* mutations represent half of these variants, the detection rate of this study is in line with the literature data.

Most mutations described in *SPAST* are predicted to disrupt the highly conserved functional domain known as the AAA cassette, which grants an effective axonal flow (39). This domain is placed between amino acids 342 and 599, which includes the predicted ATP binding and hydrolysis sites (17). No mutations have been detected in *SPAST* exon 4, which might be alternatively spliced (9, 10). Literature data show that pathogenic mutations clustered in the AAA cassette represent 77–96% of all SPAST mutations (17). In line with this, six out of nine mutations reported here are placed within AAA cassette (p.Arg460Cys, p.Pro560Leu, p.Arg450Lys, p.Arg431Ter, p.Asn405LysfsTer36, and p.Ile592SerfsTer39).

Mutations outside the AAA domain, especially missense mutations, lead spastin M1 to reduce interactions with spastin-M87, which causes a loss of microtubule-severing activity. This study reports one variant in the TM domain (p.Lys90Ter) and one in the MTBN domain (p.Lys236ProfsTer6).

Splicing events in *SPAST* are reported in approximately 10% of patients affected by inherited spastic paraplegia (12). The rate is higher in this study, where splicing mutations are reported in 4/12 of the mutations identified, thus representing 37% of patients carrying *SPAST* mutations. This frequency is likely to be underestimated since half of the disease-causing mutations in the human gene mutation database affect splicing (45, 46); however, only 16% of these mutations are placed within splicing sites (43).

This number is underestimated since it does not take into account on-canonical splice variants within the gene (missense mutations, which could represent ESE and ISE motifs), or intronic variants located more than 100 base pairs from exon–intron boundaries, which could activate cryptic (non-canonical) splice sites or alter splicing enhancer or silencer elements in introns (47).

The identification of such variants requires other approaches based on RNA analysis (48). The importance of mutations in the AAA cassette is also evident in splicing mutations. All splicing variants identified in our cohort of patients (c.1005-1delG, c.1245+1G>A, c.1414-2A>T, and c.1729-1G>C) fall in the AAA Cassette. Of these, two variants are novel, while c.1245+1G>A has already been reported (11).

Our review of literature on splicing variants in *SPAST* identified 48 splicing variants, 83.3 % are placed in the AAA Cassette (40/48), while 6.2% (3/48) are placed in the MIT motifs, and 10.4% (5/48) are placed in the MTBN motifs (Supplementary Table 2, Figure 1). Within the AAA Cassette, the splicing variants are mainly placed within all introns except the intron 10. Most of these are canonical splicing variants since they fall in the first two nucleotides outside the exons. In fact, 38 out of 48 variants are classified as class 4/5 (80%) and 10 as VoUS (20%) (Supplementary Table 2). In line with this, the splicing variants identified in the present report are canonical splicing variants and are classified as 4/5 (c.1005-1delG, c.1245+1G>A, c.1414-2A>T, and c.1729-1G>C).

In two out of four splicing variants, functional assay confirms the pathogenic mechanism suggested by *in silico* analysis, thus validating their pathogenicity. In detail, for the splicing variant c.1414-2A>T reported in Patient II:1 of Family 497 and c.1245+1G>A in Patient III:2 of Family 159, the loss of acceptor and donor site, respectively, which produces an exon-skipping proposed by *in silico* analysis is confirmed by minigene assay indicating the loss of exon 9 and 12, respectively. These data were not evaluated for c.1729-1G>C of Family 861 since the variant is present on the last exon of the gene, which does not allow setting up the minigene assay.

In Patient II:2 of Family 132, functional study deciphers the correct pathogenic mechanism caused by c.1005-1delG splicing mutations. For instance, *in silico* analysis suggests that this variant, causes a loss of acceptor site, which should have produced the skipping of exon 7. In contrast, the minigene assay followed by Sanger sequencing shows a loss of eight nucleotides of exon 7, determining the loss of frame and formation of a premature stop codon.

Functional studies, such as minigene assay or RNA analysis, can add important value for variant classification according to ACMG. Codes PS3 and BS3 can be used for “well-established” functional assays, demonstrating whether a variant has abnormal or normal gene/protein function. Although this classification does not provide guidance on how functional evidence should be evaluated, it remains relevant since the code PS3 allows classification as a pathological variant previously considered as VoUS (49–51).

These data are not crucial for canonical splicing variants since they are classified as pathological without the help of functional studies. For example, adding PS3 to the canonical splice mutations identified in this study does not change the variant classification, and functional assays remain useful to shed light on the correct pathological mechanism.

In contrast, PS3 is crucial for non-canonical splice mutations, which are often considered as VoUS, and PS3 could be useful for switching these variants to class 4 or 5 (20).

Although this study considered a small number of *SPAST* variants, and family segregation or functional analysis was performed in a few numbers of patients, this study highlights the relevance of *SPAST* in diagnosing HSP, and the high frequency of splicing mutations in the *SPAST* gene both in our cohort of patients and in patients from literature data concomitantly reviewed in the present study.

In addition, we suggest that pathological mechanisms caused by each splicing mutation could be wrongly predicted by *in silico* analysis; thus, functional assays are an essential tool to decipher the correct molecular mechanism. Although *in silico* analysis is helpful to assess potential pathogenic mechanisms, and ACMG guidelines remain essential for establishing variant pathogenicity, molecular insights such as the analysis of minigene assay are helpful to answer additional questions such as the right pathogenic mechanism caused by some splicing variants.

The conclusions of the present study remark on the need to implement molecular analysis in patients affected by spastic paraplegia to improve the identification of some specific variants and interpreting their functional relevance (Supplementary Figure 1).

## Data availability statement

The datasets presented in this article are not readily available because of ethical and privacy restrictions. Requests to access the datasets should be directed to the corresponding author.

## Ethics statement

The studies involving humans were approved by the IRCCS Neuromed Ethics Committee. The studies were conducted in accordance with the local legislation and institutional requirements. The participants provided their written informed consent to participate in this study. Written informed consent was obtained from the individual(s) for the publication of any potentially identifiable images or data included in this article.

## Author contributions

RF: Data curation, Formal analysis, Methodology, Project administration, Validation, Writing – original draft. SS: Formal analysis, Methodology, Software, Writing – original draft. AS: Conceptualization, Validation, Writing – review & editing. RC: Methodology, Validation, Writing – original draft. FA: Conceptualization, Investigation, Writing – original draft. AZ: Investigation, Writing – original draft. MC: Data curation, Formal analysis, Methodology, Supervision, Writing – original draft. AG: Data curation, Writing – original draft. MS: Writing – review & editing. AP: Conceptualization, Writing – original draft. EG: Data curation, Writing – review & editing. SZ: Conceptualization, Supervision, Writing – review & editing. FF: Supervision, Writing – review & editing. GN: Supervision, Writing – review & editing. MF: Conceptualization, Writing – original draft. CZ: Conceptualization, Investigation, Writing – original draft. GL: Conceptualization, Writing – review & editing. DC: Supervision, Writing – review & editing. SG: Project administration, Software, Supervision, Validation, Writing – review & editing.
