# Supplementary material for: Cohort analysis of novel SPAST variants in SPG4 patients and implementation of in vitro and in vivo studies to identify the pathogenic mechanism caused by splicing mutations
Source: Front Neurol. 2023 Dec 7;14:1296924. doi: 10.3389/fneur.2023.1296924 (PMC10748595; doi:10.3389/fneur.2023.1296924)
Supplement: Supplementary file 1 [file Table_1.docx]

**Table S1. Target Genes Included in the NGS Panel**

|  |  |  |  |  |  |  |  |  |  |  |  |  |  |  |
| --- | --- | --- | --- | --- | --- | --- | --- | --- | --- | --- | --- | --- | --- | --- |
| **Gene** | **MIM** | **Ref sequence** |  | **Gene** | **MIM** | **Ref sequence** |  | **Gene** | **MIM** | **Ref sequence** |  | **Gene** | **MIM** | **Ref sequence** |
| *ABCD1* | 300371 | NM_000033 |  | *CLCN1* | 118425 | NM_000083 |  | *HINT1* | 601314 | NM_005340 |  | *RAB3GAP2* | 609275 | NM_012414 |
| *AIMP1* | 603605 | NM_001142416 |  | *CLCN2* | 600570 | NM_004366 |  | *HSPD1* | 118190 | NM_002156 |  | *REEP1* | 609139 | NM_022912 |
| *ALS2* | 606352 | NM_020919 |  | *CYP2U1* | 610670 | NM_183075 |  | *HSPG2* | 142461 | NM_005529 |  | *REEP2* | 609347 | NM_016606 |
| *AMPD2* | 102771 | NM_004037 |  | *CYP7B1* | 603711 | NM_004820 |  | *KCNK18* | 613655 | NM_001291860 |  | *RTN2* | 603183 | NM_005619 |
| *AP4B1* | 607245 | NM_001253852 |  | *DDHD1* | 614603 | NM_001160148 |  | *KIF1A* | 601255 | NM_181840 |  | *SCN1A* | 182389 | NM_006920 |
| *AP4E1* | 607244 | NM_007347 |  | *DDHD2* | 615003 | NM_015214 |  | *KIF1C* | 603060 | NM_006612 |  | *SLC16A2* | 300095 | NM_006517 |
| *AP4M1* | 602296 | NM_001363671 |  | *EIF2B1* | 606686 | NM_001414 |  | *KIF5A* | 602821 | NM_004984 |  | *SLC33A1* | 603690 | NM_004733 |
| *AP4S1* | 607243 | NM_001254729 |  | *EIF2B2* | 606454 | NM_014239 |  | *L1CAM* | 308840 | NM_001278116 |  | *SPART* | 607111 | NM_001142295 |
| *AP5Z1* | 613653 | NM_014855 |  | *EIF2B3* | 606273 | NM_020365 |  | *LMNB1* | 150340 | NM_005573 |  | *SPAST* | 604277 | NM_014946 |
| *ARL6IP1* | 607669 | NM_015161 |  | *EIF2B4* | 606687 | NM_015636 |  | *LYST* | 606897 | NM_001301365 |  | *SPG11* | 610844 | NM_025137 |
| *ARSA* | 607574 | NM_000487 |  | *EIF2B5* | 603945 | NM_003907 |  | *MAG* | 159460 | NM_002361 |  | *SPG21* | 608181 | NM_016630 |
| *ARSI* | 610009 | NM_001012301 |  | *ENTPD1* | 601752 | NM_001164178 |  | *MARS* | 156560 | NM_004990 |  | *SPG7* | 602783 | NM_003119 |
| *ATL1* | 606439 | NM_015915 |  | *ERLIN1* | 611604 | NM_001320916 |  | *NIPA1* | 608145 | NM_144599 |  | *SYNE1* | 608441 | NM_182961 |
| *ATP1A2* | 182340 | NM_000702 |  | *ERLIN2* | 611605 | NM_001362878 |  | *NOTCH3* | 600276 | NM_000435 |  | *TECPR2* | 615000 | NM_014844 |
| *B4GALNT1* | 601873 | NM_001478 |  | *FA2H* | 611026 | NM_024306 |  | *NT5C2* | 600417 | NM_012229 |  | *TFG* | 602498 | NM_001195478 |
| *BICD2* | 609797 | NM_015250 |  | *FAM126A* | 610531 | NM_032581 |  | *PDGFRB* | 173410 | NM_002609 |  | *TREX1* | 606609 | NM_033629 |
| *BSCL2* | 606158 | NM_032667 |  | *FLRT1* | 604806 | NM_013280 |  | *PGAP1* | 611655 | NM_024989 |  | *TUBB4A* | 602662 | NM_001289131 |
| *C12orf65* | 613541 | NM_152269 |  | *GAD1* | 605363 | NM_000817 |  | *PLP1* | 300401 | NM_001128834 |  | *USP8* | 603158 | NM_001128610 |
| *C19orf12* | 614297 | NM_001031726 |  | *GALC* | 606890 | NM_001201401 |  | *PNPLA6* | 603197 | NM_001166111 |  | *VPS37A* | 609927 | NM_152415 |
| *CACNA1A* | 601011 | NM_023035 |  | *GBA2* | 609471 | NM_020944 |  | *POLR3A* | 614258 | NM_007055 |  | *WDR48* | 612167 | NM_001303403 |
| *CACNA1S* | 114208 | NM_000069 |  | *GJC2* | 608803 | NM_020435 |  | *POLR3B* | 614366 | NM_018082 |  | *ZFR* | 615635 | NM_016107 |
| *CCT5* | 610150 | NM_001306153 |  | *GRID2* | 602368 | NM_001510 |  | *PSAP* | 176801 | NM_001042465 |  | *ZFYVE26* | 612012 | NM_015346 |
|  |  |  |  |  |  |  |  |  |  |  |  | *ZFYVE27* | 610243 | NM_001002261 |
|  |  |  |  |  |  |  |  |  |  |  |  |  |  |  |
