# Supplementary material for: Cohort analysis of novel SPAST variants in SPG4 patients and implementation of in vitro and in vivo studies to identify the pathogenic mechanism caused by splicing mutations
Source: Front Neurol. 2023 Dec 7;14:1296924. doi: 10.3389/fneur.2023.1296924 (PMC10748595; doi:10.3389/fneur.2023.1296924)
Supplement: Supplementary file 2 [file Table_2.docx]

**Table S2. List of literature review of splicing variants identified.**

| **Splicing Variant** | **dbSNP** | **ACMG** | **Reference** |
| --- | --- | --- | --- |
| c.415+1G>A | rs1057521135 | 5 | (29) |
| c.415+1G>T | rs1057521135 | 5 | (29) |
| c.683-2A>G | [rs1553314864](https://varsome.com/variant/hg38/rs1553314864?&annotation-mode=germline) | 5 | (8) |
| c.870+3A>G | rs1553314979 | 3,4 | (31) |
| c.1004+2T>G | rs1553315240 | 5 | (8) |
| c.1004+5 G>T |  | 3,4 | (17) |
| c.1005-2A>T |  | 5 | (17) |
| c.1005-1delG |  | 5 | Our study |
| c.1098+1G>T | [rs1377020559](https://varsome.com/variant/hg38/rs1377020559?&annotation-mode=germline) | 5 | (8) |
| c.1099-1G>T |  | 5 | (17) |
| c.1173+1G>A | [rs1060502226](https://varsome.com/variant/hg38/rs1060502226?&annotation-mode=germline) | 5 | (8) |
| c.1173+1G>T |  | 5 | (17) |
| c.1245+1G>C |  | 5 | (32) |
| c.1245+1G>A |  | 5 | (33) Our study |
| c.1245+1G>T |  | 5 | (34) |
| c.1245+3G>C |  | 3,4 | (17) |
| c.1245+4_1245+ 5insA |  | 3,4 | (17) |
| c.1245+5G>T |  | 3,4 | (17) |
| c.1245+5G>A | rs1553317049 | 3,4 | (17);(35) |
| c.1246-1G>T |  | 5 | (17) |
| c.1413+1G>T | [rs1553318276](https://varsome.com/variant/hg38/rs1553318276?&annotation-mode=germline) | 5 | (36) |
| c.1413+2T>A |  | 5 | (37) |
| c.1413+2T>G | [rs1553318280](https://varsome.com/variant/hg38/rs1553318280?&annotation-mode=germline) | 5 | (38) |
| c.1413+3_1413+ 6delAAGT | [rs570685843](https://varsome.com/variant/hg38/rs570685843?&annotation-mode=germline) | 4 | (15) |
| c.1413 + 1_1413+4delGTA |  | 4 | (39) |
| c.1413+5G>A | [rs1553318282](https://varsome.com/variant/hg38/rs1553318282?&annotation-mode=germline) | 3,4 | (8) |
| c.1414-1G>T |  | 5 | (17) |
| c.1414-2A>T |  | 5 | Our study |
| c.1493+18G>T | rs189961829 | 1 | (17) |
| c.1493+2_1493+5 delTAGG | [rs1558337180](https://varsome.com/variant/hg38/rs1558337180?&annotation-mode=germline) | 5 | (52) |
| c.1494-1G>A | [rs1315245986](https://varsome.com/variant/hg38/rs1315245986?&annotation-mode=germline) | 5 | (17) |
| c.1494-1G>C | [rs1315245986](https://varsome.com/variant/hg38/rs1315245986?&annotation-mode=germline) | 5 | (17) |
| c.1494-2A>G | [rs121808125](https://varsome.com/variant/hg38/rs1218081251?&annotation-mode=germline) | 5 | (40) |
| c.1536+2T>G |  | 5 | (39) |
| c.1536+1G>T | [rs1553319095](https://varsome.com/variant/hg38/rs1553319095?&annotation-mode=germline) | 5 | (8) |
| c.1537-8T>G |  | 3 | (20) |
| c.1616+1dup |  | 5 | (41) |
| c.1616+2T>C |  | 5 | (17) |
| c.1616-2T>A |  | 5 | (17) |
| c.1617-2A>G |  | 5 | (42) |
| c.1687+1G>A | [rs1553319556](https://varsome.com/variant/hg38/rs1553319556?&annotation-mode=germline) | 5 | (8) |
| c.1687+2T>C |  | 5 | (17) |
| c.1687+5G>C |  | 3,4 | (17) |
| c.1688-1G>A | [rs1573174147](https://varsome.com/variant/hg38/rs1573174147?&annotation-mode=germline) | 5 | (43) |
| c.1688-1G>C |  | 5 | (45) |
| c.1728+1G>A | [rs587777754](https://varsome.com/variant/hg38/rs587777754?&annotation-mode=germline) | 5 | (8) |
| c.1729-1G>C | [rs1064793976](https://varsome.com/variant/hg38/rs1064793976?&annotation-mode=germline) | 5 | Our study |
| c.1729-1G>A | [rs1064793976](https://varsome.com/variant/hg38/rs1064793976?&annotation-mode=germline) | 5 | (39) |
